# Matrix metalloproteinases and diabetic foot ulcers: the ratio of MMP-1 to TIMP-1 is a predictor of wound healing

**DOI:** 10.1111/j.1464-5491.2008.02414.x

**Published:** 2008-04-01

**Authors:** M Muller, C Trocme, B Lardy, F Morel, S Halimi, P Y Benhamou

**Affiliations:** Department of Nephrology and Endocrinology, University Hospital Grenoble, France; * Enzymology Laboratory/GREPI TIMC-Imag CNRS 5525, Department of Cell Biology and Pathology, University Hospital Grenoble, France

**Keywords:** diabetic foot ulcer, wound healing, matrix metalloproteinase

## Abstract

**Aims:**

Matrix metalloproteinases (MMPs) play a major role in wound healing: they can degrade all components of the extracellular matrix. In diabetic foot ulcers there is an excess of MMPs and a decrease of the tissue inhibitors of MMPs (TIMPs). This imbalance is probably one cause of impaired healing. However, little is known about changes in MMPs during wound healing.

**Methods:**

Sixteen patients with neuropathic diabetic foot ulcers participated. Wound fluid was collected regularly during the 12-week follow-up period, for measurement of MMP-1, MMP-2, MMP-8, MMP-9 and TIMP-1. Results were analysed by the degree of wound healing: good healers (defined by a reduction of at least 82% in initial wound surface at 4 weeks) and poor healers (reduction of less than 82% in wound surface at 4 weeks).

**Results:**

In good healers, levels of MMP-8 and -9 secreted by inflammatory cells decreased earlier. The initial levels of MMP-1 were similar in good and poor healers (*P* = 0.1) but rose significantly at week 2 in good healers (*P* = 0.039). There was a significant correlation between a high ratio of MMP-1/TIMP-1 and good healing (*r* = 0.65, *P* = 0.008). Receiver Operator Curve (ROC) analysis showed that an MMP-1/TIMP-1 ratio of 0.39 best predicted wound healing (sensitivity = 71%, specificity = 87.5%).

**Conclusions:**

A high level of MMP-1 seems essential to wound healing, while an excess of MMP-8 and -9 is deleterious, and could be a target for new topical treatments. The MMP-1/TIMP-1 ratio is a predictor of wound healing in diabetic foot ulcers.

Diabet. Med. 25, 419–426 (2008)

## Introduction

Diabetic foot ulcers pose a major public health problem: they are the leading cause of non-traumatic amputation in developed countries. The medical treatment of these chronic wounds remains a challenge for clinicians. Better understanding of the pathophysiology of these chronic wounds should improve their treatment.

MMPs are a family of zinc endopeptidases capable of degrading all the components of the extracellular matrix [1–3]. They are key players in every phase of the healing process: they eliminate damaged protein, destroy the provisional extracellular matrix, facilitate migration to the centre of the wound, remodel the granulation tissue, probably control angiogenesis [4] and also regulate the activity of some growth factors. MMPs are divided into sub-groups depending on the specificity of their substrates. Collagenases (MMP-1 and -8) and gelatinases (MMP-2 and -9) play a major role in the healing process. Their activity is specifically inhibited by TIMPs, four of which have been identified. With few exceptions, all the TIMPs are capable of inhibiting all the MMPs, although TIMP-1 has a higher affinity for MMP-1 and -9 and TIMP-2 for MMP-2 and -8.

Although essential in every phase of the healing process, MMPs are finely regulated and a successful healing process is dependent on a rigorous spatial and temporal pattern of expression [5–7]. Whereas MMP levels decrease through the normal wound-healing process, chronic wounds contain a significantly higher level of proteases and pro-inflammatory cytokines (tumour necrosis factor-α and interleukin-1), as well as lower levels of growth factors [8–11]. In particular, collagenase [12,13] and gelatinase [14] activity is increased in chronic wounds, while the TIMP-1 level is reduced compared to acute wounds [12,14,15].

These data have led to a unifying pathophysiological hypothesis: the inflammatory phase of healing is exaggerated in chronic wounds (venous ulcers, pressure ulcers, diabetic foot ulcers) [3,16] leading to an excess of proteases and inflammatory cytokines released by neutrophils and macrophages [17,18]. Thus, the excess of proteases and MMPs in chronic wounds is probably one of the reasons for poor healing by breaking down too many components of the extracellular matrix and by inhibiting growth factors that are essential for tissue synthesis [3,11].

Few studies have measured MMP activity in wound fluid from diabetic foot ulcers. Lobman *et al.* found that levels of MMP-1, MMP-8, MMP-9 and activated MMP-2 were significantly higher in diabetic foot ulcers and the level of TIMP-2 significantly lower than in acute wounds from non-diabetic patients [19]. Likewise, there are very little data concerning the change in MMP levels during the healing of chronic diabetic foot ulcers.

The primary objective of this study was to describe changes in MMP and TIMP levels during healing in diabetic foot ulcers, and thus to improve our scant knowledge of this process. The secondary objective was to search for any correlation between changes in MMP and TIMP levels and wound healing, in order to find possible predictors of healing.

## Subjects and methods

### Patients

This prospective pilot study recruited 16 consecutive Type 2 diabetic patients aged over 40 years from the Diabetology Department of the Grenoble University Hospital from May 2005 to June 2006. Patients were eligible if they had: (1) a diabetic foot ulcer rated 1 to 3, stage A according to the University of Texas Wound Classification (not infected and no severe arteriopathy); (2) a chronic wound (at least 30 days’ duration); (3) a wound area larger than 0.5 cm^2^ at inclusion. Patients were ineligible if they had an infected wound (based on the International Consensus on The Diabetic Foot criteria 2003) or arteriopathy of the lower limbs, characterized either by absence of posterior tibial and pedal pulses or by an ankle/brachial index < 0.9. We excluded soft tissue infections, because bacteria may secrete MMPs. We did not exclude osteomyelitis because chronic osteomyelitis in particular may not necessarily be associated with soft-tissue infection.

### Study design

The study was approved by the institutional review board (Person Protection Committee CPP of Grenoble University Hospital) and each patient gave written informed consent. At each visit [week 0 (W0), W1, W2, W4, W8 and W12], the wound area was measured using a numeric photograph and appropriate software (Mouseyes®, Salford, UK; http://www.hop.man.ac.uk/staff/rtaylor). Two samples of wound fluid were collected using sterile absorbent paper strips placed on the edges of the wound for 5 min, in order to measure MMP-1, -2, -8, -9 and TIMP-1 levels. This method for the measurement of MMPs has been validated for other sample types, particularly for tears [20].

The local treatment was the same for all wounds. We followed the protocol used for patients presenting with diabetic foot ulcers in our department (local care given by a nurse every 2 days) and choice of the dressing according to our local protocol (briefly, a wet dressing for dry wounds and an absorbent wound dressing for exudative wounds). No dressing known to interfere with MMP levels (such as Beclapermine or Promogran) was used.

### Biological parameters

The assays of MMP-1, -2, -8 and -9 and TIMP-1 were performed at the Enzymology Laboratory (Grenoble University Hospital).

Protein elution from the Shirmer strips was performed by stirring the strips in 1 ml of buffer (50 mM Tris, 50 mM NaCl, 0.05% Brij 35, pH 7.6) for at least 2 h at +4°C.

The levels of MMP-2 and -9 were measured using zymography [20]. Briefly, proteins were separated on an SDS-PAGE gel copolymerized with 0.5 mg/ml gelatine. After incubation in a buffer to activate the enzyme, the gel is stained with Coomassie Blue (Sigma, Saint Quentin Fallavier, France): proteins with gelatinolytic activity are thus detected as unstained bands. The quantity of enzyme is assessed by densitometry of the lysis bands, the area under the curve thus obtained being referred to a standard scale of purified gelatinase. This method quantifies both latent and activated forms of MMP-2 and -9, and, by addition, the total concentration of each gelatinase.

The concentrations of MMP-1, MMP-8 and TIMP-1 were measured using an ELISA technique (R&D Systems, Lille, France for MMP-1, Amersham, Orsay, France for MMP-8 and Oncogene Research, VWR International, Fontenay sous Bois, France for TIMP-1). To avoid possible variations caused by the amount of fluid collected, protein concentration was assayed using the Pierce method [21] and the concentrations of MMPs and TIMP-1 were expressed as pg/µg protein.

### Statistical analysis

The statistical software program SSPS® 11.0 (SPSS Inc., Chicago, IL, USA) was used for statistical analyses, performed by the Clinical Investigation Centre (CIC INSERM, Grenoble University Hospital). A threshold of α = 0.05% was used for all statistical tests.

The 16 patients were divided into two sub-groups according to the rate of ulcer healing. In a recent large prospective trial [22], the mean percentage reduction in wound area over a 4-week period was 82%[95% confidence interval (CI) 70–94] in the group of patients whose wound had completely healed at the end of the 12-week follow-up period. Therefore in our study, a decrease in wound area of at least 82% at 4 weeks defined the ‘good healers’ and a decrease in wound area of less than 82% at 4 weeks defined the ‘poor healers’.

Quantitative parameters were expressed as the median and 25th and 75th percentiles. Qualitative parameters were expressed in effectives and percentages. Non-parametric tests were used: the Mann–Whitney or the Wilcoxon test and the Chi-squared or Fisher test. The Spearman test was performed to detect correlations between percentage decrease in wound area and changes in the MMPs or TIMPs levels. However, the retrospective division into good and poor healers was not used for detecting these correlations. ROC analysis was used to determine whether the parameters had any predictive value with respect to wound healing. Correlations between MMPs and healing, as well as the ROC curves, were established using a population of 15 patients. One patient was excluded because he underwent amputation at W8.

## Results

### Clinical data

**FIGURE 1:**
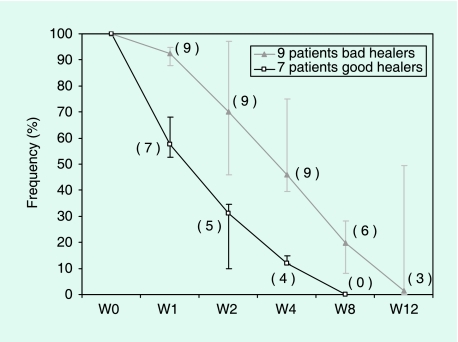
Wound area during the 12-week follow-up, expressed as a percentage of the initial area. The values correspond to medians for each group, with 25th and 75th percentiles. The number of patients whose wound is not completely healed is given in parentheses for each visit and each group. By week 8 7/7 patients had healed in the good healer group, whereas by week 12 3/9 patients had not healed in the poor healer group.

Sixteen Type 2 diabetic patients [mean age 64 years (range 47–84 years), 15 men and 1 woman] participated. The main characteristics of the two sub-groups (seven good healers and nine poor healers) are summarized in Table 1. The frequency of diabetes complications (neuropathy, retinopathy, nephropathy, arteriopathy of the lower limbs) was similar in the two groups. However, four patients who satisfied the inclusion criteria (at least one distal pulse) were later shown, by arterial Doppler, to have developed an IPS < 0.9 (respectively 0.4/0.5/0.8 and 0.84), suggesting arterial disease. Figure 1 shows the change in wound size in both groups during the 12 weeks of follow-up. All patients completed all the follow-up visits unless their wound healed completely before the end of the study. In the good healers group, all the wounds had completely closed before W12.

**Table 1:** Patients'characteristics at inclusion

	Good healers (*n* = 7)	Poor healers (*n* = 9)
Age (years)	60 (58–70)	61 (58–77)
Sex (M/F)	6/1	9/0
Duration of diabetes (years)	10 (10–25)	15 (10–25)
HbA_1c_ (%)	12.4 (6.3–12.5)	9.1 (7.6–9.6)
Wound duration (months)	1 (1–1)	5* (2–6)
Initial wound area (cm^2^)	1.22 (0.95–1.29)	3.62* (2.23–5.1)
Initial wound depth (mm)	2 (1–2)	3.5 (2–5)
Frequency of osteomyelitis	2/7 (29%)	2/9 (22%)
ABi	1.10 (1.1–1.2)	1.10 (0.7–1.2)

HbA_1c_, glycated haemoglobin; ABi, ankle brachial index.

* *P* (good vs. poor healers) < 0.05.

Values are expressed as median (25–75th percentile).

Oral antibiotics were given to the two patients (one in each group) with proven osteomyelitis.

No patients received tetracyclines or chemically modified tetracyclines, the only antibiotics known to influence MMP levels.

### Kinetics of MMP expression during the wound-healing process

**FIGURE 2:**
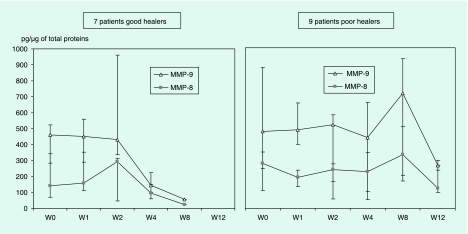
Levels of MMP-8 and MMP-9 for good and poor healers during the 12-week follow-up period (results are expressed as medians with 25th and 75th percentiles).

**FIGURE 3:**
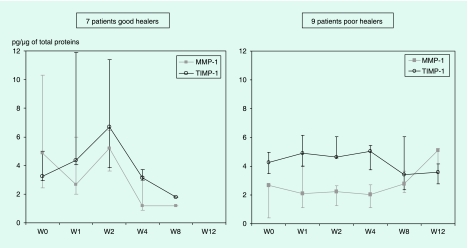
Levels of MMP-1 and TIMP-1 for good and poor healers during the 12-week follow-up period (results are expressed as medians with 25th and 75th percentiles).

Initial levels of the different MMPs are shown in Table 2. There were no significant differences in any parameter between the two groups. During the 12 weeks of follow-up, the changing profile of MMPs differed between groups (Figs 2 and 3). Figure 2 shows that MMP-8 and -9 levels in good healers remained stable between W0 and W2, before starting to decrease from W2 onwards. The change in MMP-9 levels between W0 and W4 did not reach significance (*P* = 0.14). In contrast, in poor healers MMP-8 and -9 levels remained constant throughout the follow-up period. Moreover (Table 2 and Fig. 3), the level of MMP-1 was significantly higher at W0 in good healers compared to poor healers at week 2 [4.78 pg/µg of protein (CI 1.9–7.65) vs. 2.27 pg/µg (CI 1.24–0.29); *P* = 0.039]. MMP-1 level in good healers appeared to diminish during the study whereas it remained stable in poor healers. Over the same follow-up period, there was no significant difference in the TIMP-1 level between the two groups.

**Table 2:** Levels of matrix metalloproteinases at inclusion (week 0 visit) in pg/µg protein

	Good healers (*n* = 7)	Poor healers (*n* = 9)
MMP-1	4.88*	2.66
	(2.43–10.32)	(9–2.69)
Total MMP-2	22.89	42.22
	(12.4–37.78)	(23.63–57.46)
Activated MMP-2	5.41	5.75
	(0–9.82)	(4.95–7.88)
MMP-8	140.44	279.63
	(70.8–342.2)	(109.5–350)
Total MMP-9	461.6	479.4
	(282–524)	(246.4–876.6)
Activated MMP-9	0	20.85†
	(0–7.16)	(0–81.2)
TIMP-1	3.25	4.26
	(2.93–5.01)	(3.47–4.96)

Values are expressed as median (25–75th percentile).

MMP, matrix metalloproteinase; TIMP, tissue inhibitor of matrix metalloproteinase.

* *P* = 0.1.

† *P* = 0.068.

MMP-2 level did not differ between the groups during the study (Table 2 and data not shown), and no particular pattern of change could be characterized.

### Predictors of healing

The ratio between MMP and TIMP levels in wound fluid better reflects the proteolytic environment of the wound than the two measurements taken separately. The MMP-1/TIMP-1 ratio was not significantly different at W0 in good healers compared to poor healers (*P* = 0.064). However, the percentage reduction in wound area at W4 correlated positively with the MMP-1/TIMP-1 ratio at W0 (*r* = 0.65; *P* = 0.008) and at W2 (*r* = 0.69, *P* = 0.009). Hence the MMP-1/TIMP-1 ratio at W0 is a prognostic indicator of complete healing at the end of the 12-week follow-up period.

**FIGURE 4:**
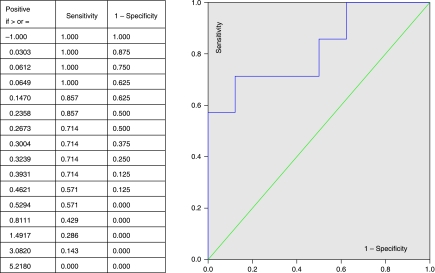
The ratio of MMP-1/TIMP-1 is a predictive factor for healing. The ROC analysis gives an area under the curve of 0.821 [confidence interval (CI) 0.6–1.04]. A ratio of 0.39 at week 0 has a sensitivity of 71% and a specificity of 87.5% for detecting a wound area reduction of at least 82% at week 4 (and thus predicting wound healing at week 12).

The ROC curve showed that an MMP-1/TIMP-1 ratio at W0 of 0.39 best predicts a reduction in wound area of at least 82% at W4—i.e. predicts complete wound healing at W12—with a sensitivity of 71% and a specificity of 87.5% (Fig. 4). Logistic regression analysis showed that this ratio at W0 is a predictive factor *a priori* independent of the wound area and depth. However, in view of the small number of patients, the results from this multivariate model should be treated with caution.

## Discussion

Remodelling of the extracellular matrix occurs in various physiological processes (post-partum uterine involution, ovulation, embryogenesis, odontogenesis, bone remodelling, cutaneous healing etc.); consequently, its synthesis and destruction is strictly regulated [23]. MMPs are the main protease family involved in this regulation. A disorder in the synthesis of MMPs and/or alteration in the enzymes/inhibitors balance have been shown in several pathological processes characterized by disorganization of the extracellular matrix, such as rheumatoid arthritis, formation of atheroma plaques, tumour development, metastatic dissemination and chronic wounds [24].

Chronic ulcers are probably caused by an exaggeration of their inflammatory phase [3,16]. This is supported by many studies where levels of MMPs were higher in the exudates of chronic wounds than in those of acute wounds, as in diabetic foot ulcers [3,19]. However, it has been more assumed than really shown that the ultimate healing of diabetic foot ulcers is accompanied by a decrease in MMP levels. Longitudinal studies of the change in MMP levels over the whole period of the healing process were needed. In other respects, although MMPs are deleterious when in excess, they are nevertheless indispensable for good healing. This has been demonstrated by studies in which topical broad spectrum inhibitors of MMPs [GM 6001 (Ilomostat)] caused prolonged healing times compared to a placebo [25,26]. Such negative results suggest that each MMP plays a specific role in the healing process and that our knowledge of the pathophysiology of chronic wounds is incomplete. In particular the exact functions of the various MMPs need to be explored.

Our study focused on a carefully selected population. We wished to avoid contamination with cytokines or MMPs produced by bacteria. Therefore, soft-tissue infection was an exclusion criterion. Although grade 3 ulcers (UT) were indeed included in our study (two patients in each group), osteomyelitis is not necessarily associated with soft-tissue infection. Therefore we consider that levels of inflammatory cytokines were the same in wounds with osteomyelitis as in wounds without osteomyelitis.

There is currently no consensus as to the best method to measure MMP activity in wounds. The two usual techniques consist of collecting the wound fluid after occlusion with a dressing or to take a biopsy at the wound edges. However major differences in MMP concentration were observed between these two measurement techniques [16]. We chose to study local expression of MMPs in wound fluid, firstly because it is accepted that fluid composition reflects the microenvironment of the wound (with a mixture of tissue and serum proteins), and secondly because biopsy could be harmful to small-size wounds in diabetes. Samples were collected using absorbent strips (Schirmer strips), which absorb the fluid by capillary action. These sterile strips have been previously used in ophthalmology to measure MMP-2 and -9 concentrations in the tears of patients with corneal grafts [20]. They can be used in outpatient studies because of the rapidity of the sampling. Moreover, MMP concentrations so measured are comparable to those of other studies undertaken on wound fluids and expressed in the same units (pg/µg of protein) [10,16].

In our study, we described changes in MMP levels during 12 weeks follow-up in patients with homogeneous diabetic foot ulcers. We showed two main differences between a group with good healing prognosis and a group with poor prognosis.

Firstly, there is a statistically significant relationship between the MMP-1/TIMP-1 ratio at study entry and good healing. Indeed, MMP-1 is the major collagenase implicated in wound healing: it has been shown that its specific proteolysis of type I collagen (an essential component of the dermis) is indispensable for keratinocyte migration and thus re-epidermization [27,28]. We hypothesize that the higher level of MMP-1 in good healers is favourable by permitting the proliferative phase to be completed. Furthermore, it has been suggested that an adapted regulation of MMP-1 is essential to the proper progression of the healing process [2]. The rise in TIMP-1 between W0 and W2 suggests regulation of MMP-1 activity for a correct healing.

Secondly, there is a decrease in the MMP-8 and -9 levels from the second week after study entry in good healers, but not in poor healers. This drop of both MMPs produced by inflammatory cells suggests a process of decline in the inflammatory phase whilst healing proceeds, allowing the continuation of the healing process to the next phase of proliferation. In the same way, the higher level of activated MMP-9 in the poor healer group supports the idea that an elevated level of some MMPs (those secreted by inflammatory cells) is harmful to healing. Likewise, Ladwig *et al.* described a higher level of activated MMP-9 in a group of poorly healing pressure ulcers compared to a group of good healers [16]. In their 56 patients, the MMP-9/TIMP-1 ratio was positively correlated with poor healing, which underlies the deleterious effect of an MMP-9 excess in chronic wounds. Interestingly, Lobmann *et al.* found that the MMP-9/TIMP-2 ratio was significantly reduced in a group of diabetic patients exhibiting a more rapid healing rate when treated with a protease modulating matrix. However, TIMP-2 seems less relevant as it inhibits preferentially MMP-2 [29]. We also measured the MMP-9/TIMP-1 ratio in our study, as well as the MMP-8/TIMP-1 ratio, but found no statistical difference. We consider it biologically relevant that MMP-1 was the predictive factor for wound healing because MMP-1 is implicated in the migration of keratinocytes, while MMP-8 and MMP-9 are synthesized by inflammatory cells.

Although MMP-8 and -9 are expressed at a level 10–100 times higher than other MMPs [12], MMP-1 and TIMP-1 are localized in the same area of the cutaneous tissue because they are both synthesized by the same cell type (keratinocytes and fibroblasts) [10,13]. This permits the preferential inhibition of MMP-1, particularly compared to MMP-9.

Lastly, the role of MMP-2 is not clear. As mentioned previously [16], the slight variations in its levels (because of its constitutive expression in cutaneous tissue) suggest that MMP-2 plays only a minor role in chronic wounds. Because MMP-2 is secreted by fibroblasts, produced mainly during the proliferative phase, we hypothesize that its role is less relevant in chronic wounds, where the inflammatory phase predominates.

The principle weakness of the current study is the relatively small number of patients and therefore the low statistical power of our results. Another issue concerns our two populations that differ in terms of the wound surface area and duration—two potential predictive factors for wound healing. To control for this, we used a multivariate analysis, which confirms that the MMP-1/TIMP-1 ratio is a predictive factor independent of known clinical markers.

Nevertheless, these results allow us to formulate several hypotheses. Firstly, the MMP-1/TIMP-1 ratio measured on admission of a diabetic patient with a foot ulcer is a predictor of healing and may allow treatment to be adapted accordingly. Secondly, all the MMPs present in excess in diabetic foot ulcers are not necessarily harmful to healing. Thus the development of new topical treatments that act on MMPs should target specific inhibitors of certain MMPs—probably those secreted by inflammatory cells (MMP-8 and -9).

In conclusion, we have performed the first longitudinal study describing the kinetics of expression of MMPs during the healing process of chronic diabetic foot ulcers. This study provides evidence for a role of the MMP-1/TIMP-1 ratio in the prediction of healing.

## Competing interests

None to declare.

## Abbreviations

ABi: ankle brachial index
ELISA: enzyme-linked immunosorbent assay
MMP: matrix metalloproteinase
ROC: receiver operator curve
TIMP: tissue inhibitor of matrix metalloproteinase

## References

[1] Armstrong DG, Jude EB (2002). The role of matrix metalloproteinases in wound healing. J Am Podiatr Med Assoc.

[2] Ravanti L, Kahari VM (2000). Matrix metalloproteinases in wound repair (Review). Int J Mol Med.

[3] Lobmann R, Schultz G, Lehnert H (2005). Proteases and the diabetic foot syndrome: mechanisms and therapeutic implications. Diabetes Care.

[4] Patterson BC, Sang QA (1997). Angiostatin-converting enzyme activities of human matrilysin (MMP-7) and gelatinase B/type IV collagenase (MMP-9). J Biol Chem.

[5] Soo C, Shaw WW, Zhang X, Longaker MT, Howard EW, Ting K (2000). Differential expression of matrix metalloproteinases and their tissue-derived inhibitors in cutaneous wound repair. Plast Reconstr Surg.

[6] Moses MA, Marikovsky M, Harper JW (1996). Temporal study of the activity of matrix metalloproteinases and their endogenous inhibitors during wound healing. J Cell Biochem.

[7] Baker EA, Leaper DJ (2003). Profile of matrix metalloproteinases and their tissue inhibitors in intraperitoneal drainage fluid: relationship to wound healing. Wound Rep Reg.

[8] Trengove NJ, Stacey MC, Macauley S, Bennett N, Gibson J, Burslem F (1999). Analysis of the acute and chronic wounds environments: the role of proteases and their inhibitors. Wound Rep Reg.

[9] Wysocki AB, Staiano-Coico L, Grinnell F (1993). Wound fluid from chronic leg ulcers contain elevated levels of metalloproteinases MMP-2 and MMP-9. J Invest Dermatol.

[10] Yager DR, Zhang LY, Liang HX, Diegelmann RF, Cohen IK (1996). Wound fluids from human pressure ulcers contain elevated matrix metalloproteinase levels and activity compared to surgical wound fluids. J Invest Dermatol.

[11] Yager DR, Stephen MC, Ward SI, Olutoye OO, Diegelmann RF, Cohen IK (1997). Ability of chronic wound fluids to degrade peptide growth factors is associated with increased levels of elastase activity and diminished levels of proteinase inhibitors. Wound Rep Reg.

[12] Nwomeh BC, Liang HX, Cohen IK, Yager DR (1999). MMP-8 is the predominant collagenase in healing wounds and nonhealing ulcers. J Surg Res.

[13] Vaalamo M, Mattila L, Johansson N, Kariniemi AL, Karjalainen-Lindsberg ML, Kahari VM (1997). Distinct populations of stromal cells express collagenase-3 (MMP-13) and collagenase-1 (MMP-1) in chronic ulcers but not in normally healing wounds. J Invest Dermatol.

[14] Bullen EC, Longaker MT, Updike DL, Benton R, Ladin D, Hou Z (1995). Tissue inhibitor of metalloproteinases-1 is decreased and activated gelatinases are increased in chronic wounds. J Invest Dermatol.

[15] Vaalamo M, Weckroth M, Puolakkainen P, Kere J, Saarinen P, Lauharanta J (1996). Patterns of matrix metalloproteinase and TIMP-1 expression in chronic and normally healing human cutaneous wounds. Br J Dermatol.

[16] Ladwig GP, Robson MC, Liu R, Kuhn A, Muir DF, Schulz GS (2002). Ratios of activated matrix metalloproteinase-9 to tissue inhibitor of matrix metalloproteinase-1 in wound fluids are inversely correlated with healing of pressure ulcers. Wound Rep Reg.

[17] Trengove NJ, Bielefeldt-Ohmann H, Stacey MC (2000). Mitogenic activity and cytokine levels in non-healing and healing chronic leg ulcers. Wound Rep Reg.

[18] Piaggesi A, Viacava P, Rizzo L, Naccarato G, Baccetti F, Romanelli M (2003). Semiquantitative analysis of the histopathological features of the neuropathic foot ulcer. Diabetes Care.

[19] Lobmann R, Ambrosch A, Schultz G, Waldmann K, Schiweck S, Lehnert H (2002). Expression of matrix-metalloproteinase and their inhibitors in the wounds of diabetic and non-diabetic patients. Diabetologia.

[20] Barro C, Romanet JP, Fdili A, Guillot M, Morel F Gelatinase concentration in tears of corneal-grafted patients. Current Eye Research.

[21] Smith PK, Krohn RI, Hermanson GT, Mallia AK, Gartner FH, Provenzano MD (1985). Measurement of protein using bicinchoninic acid. Anal Biochem.

[22] Sheehan P, Jones P, Caselli A, Giurini JM, Veves A (2003). Percent change in wound area of diabetic foot ulcers over a 4-week period is a robust predictor of complete healing in a 12-week prospective trial. Diabetes Care.

[23] Bourd-Boittin K, Fanchon S, Septier D, Menashi S, Goldberg M (2004). Métalloprotéases et inhibiteurs de métalloprotéases au cours de l'odontogénèse. Rôle biologique des sous-produits de dégradation matricielle. Les Cahiers de l'ADF.

[24] Nalbone G, Alessi MC, Juhan-Vague I (2001). Système fibrinolytique, métalloprotéases et pathologie vasculaire. Médecine/Sciences.

[25] Ågren M (1999). Matrix metalloproteinases (MMPs) are required for re-epithelialization of cutaneous wounds. Arch Dermatol Res.

[26] Ågren M, Mirastschijski U, Karlsmark T, Saarialho-Kere U (2001). Topical synthetic inhibitor of matrix metalloproteinases delays epidermal regeneration of human wounds. Exp Dermatol.

[27] Stamenkovic I (2003). Extracellular matrix remodelling: the role of matrix metalloproteinases. J Pathol.

[28] Pilcher BK, Dumin JA, Sudbeck BD, Krane SM, Welgus HG, Parks WC (1997). The activity of Collagenase-1 is required for keratinocyte migration on a type 1 collagen matrix. J Cell Biol.

[29] Lobmann R, Zemlin C, Motzkau M, Reschke K, Lehnert H (2006). Expression of matrix metalloproteinases and growth factors in diabetic foot wounds treated with a protease absorbent dressing. J Diabetes Complications.

